# 175. An Open-Label Phase 1 Clinical Trial Demonstrating Improved Immune Responses to Norovirus Strains GI.1 and GII.4 from a Second Generation Oral Bivalent Vaccine Candidate

**DOI:** 10.1093/ofid/ofaf695.005

**Published:** 2026-01-11

**Authors:** Nicholas J Bennett, Becca A Flitter, Colin A Lester, Nick P D’Amato, Maria D Apkarian, Sean N Tucker, James F Cummings

**Affiliations:** Vaxart Inc, PONTE VEDRA, Florida; Vaxart Inc, PONTE VEDRA, Florida; Vaxart, Inc., South San Francisco, California; Vaxart Inc., South San Francisco, California; Vaxart Inc., South San Francisco, California; Vaxart, South San Francisco, California; VAXART, South San Francisco, California

## Abstract

**Background:**

Norovirus is a pathogen of global importance, responsible for hundreds of thousands of deaths and economic costs of tens of billions of dollars. No licensed vaccine to norovirus currently exists. In a previous placebo-controlled phase 2b norovirus human challenge study of our first generation oral vaccine candidate (FGV), machine learning analysis identified norovirus blocking antibody assay (NBAA) as a critical correlate of protection. Small modifications to the non-coding regions and the antigen sequence in our second generation vaccine candidate (SGV) resulted in improved antigen expression. A human study was performed to test for improved immunogenicity.

**Figure:**
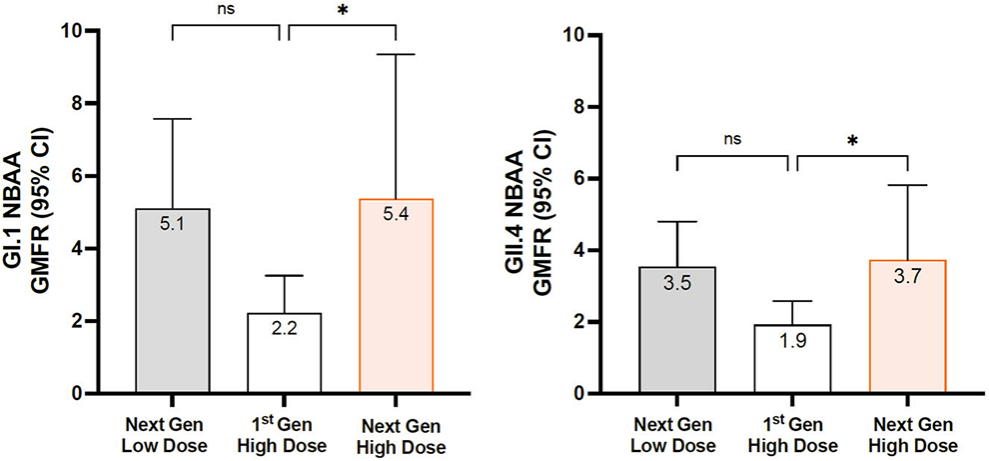
Norovirus Blocking Antibody Assay Results Between Arms for First and Second Generation Vaccine Candidates Low dose: 1e10 of each strain. High dose: 1e11 of each strain. *: statistically significant, p < 0.05. ns: not statistically significant. GMFR: Geometric Mean Fold Rise. NBAA: Norovirus Blocking Antibody Assay. Fold-rise measured between pre-vaccination baseline and 28-days post-vaccination.

**Methods:**

An open-label phase 1 clinical trial was conducted in 60 healthy adult volunteers comparing two dose levels of bivalent SGV (1e10 or 1e11 of each strain) to FGV (1e11 of each strain). 20 subjects in each study arm were enrolled sequentially to receive low-dose SGV, high-dose FGV, or high dose SGV respectively. Subjects were followed for solicited symptoms for 7 days, and unsolicited adverse events (AE) for 28 days. NBAA Geometric Mean Fold Rise (GMFR) and cross-reactive serum antibody by multiplex MSD were measured.

**Results:**

All confirmed solicited symptoms were of mild to moderate severity and the number and severity of symptoms were similar between study arms. Overall, the most frequently reported solicited symptom was headache, reported in 16.7% of participants. Similar numbers of participants in each arm reported unsolicited AE, which were all mild or moderate in severity, with no evidence of a dose-dependent effect. No serious AE or adverse events of special interest were reported. SGV administration improved GI.1 and GII.4 NBAA titers compared to the FGV. A GMFR of 5.1 of GI.1 was observed for Arm 1 and 5.4 for Arm 3, compared to the Arm 2 GMFR of 2.2. Similarly, a GMFR of 3.5 in Arm 1 and 3.7 in Arm 3 for GII.4 was improved over 1.9 observed in Arm 2. Statistical significance of < 0.05 was achieved for the high dose SGV for both strains compared to FGV. Lastly, SGV administration induced improved cross-reactive serum antibody responses to multiple genotypes.

**Conclusion:**

Our oral bivalent norovirus SGV demonstrated improved immune responses compared to FGV, with no notable safety concerns. A larger Phase 2 study is planned.

**Disclosures:**

All Authors: No reported disclosures

